# Green Synthesis and Antimicrobial Activities of Silver Nanoparticles Using *Calotropis gigantea* from Ie Seu-Um Geothermal Area, Aceh Province, Indonesia

**DOI:** 10.3390/molecules27165310

**Published:** 2022-08-20

**Authors:** Pati Kemala, Rinaldi Idroes, Khairan Khairan, Muliadi Ramli, Zulkarnain Jalil, Ghazi Mauer Idroes, Trina Ekawati Tallei, Zuchra Helwani, Eka Safitri, Muhammad Iqhrammullah, Rosnani Nasution

**Affiliations:** 1 Graduate School of Mathematics and Applied Sciences, Universitas Syiah Kuala, Banda Aceh 23111, Indonesia; 2 Department of Chemistry, Faculty of Mathematics and Natural Sciences, Universitas Syiah Kuala, Banda Aceh 23111, Indonesia; 3 Department of Pharmacy, Faculty of Mathematics and Natural Sciences, Universitas Syiah Kuala, Banda Aceh 23111, Indonesia; 4 Herbal Medicine Research Center, Universitas Syiah Kuala, Banda Aceh 23111, Indonesia; 5 Department of Physics, Faculty of Mathematics and Natural Sciences, Universitas Syiah Kuala, Banda Aceh 23111, Indonesia; 6 Department of Chemical Engineering, Faculty of Engineering, Universitas Syiah Kuala, Banda Aceh 23111, Indonesia; 7 Department of Biology, Faculty of Mathematics and Natural Sciences, Sam Ratulangi University, Manado 95115, Indonesia; 8 Department of Chemical Engineering, Faculty of Engineering, Universitas Riau, Pekanbaru 28293, Indonesia; 9 Department of Life Sciences and Chemistry, Jacobs University Bremen, Campus Ring 1, 28759 Bremen, Germany

**Keywords:** silver nanoparticles, geothermal manifestation, Ie Seu-Um, *C. gigantea*, antimicrobial activities

## Abstract

Herein, we report our success synthesizing silver nanoparticles (AgNPs) using aqueous extracts from the leaves and flowers of *Calotropis gigantea* growing in the geothermal manifestation Ie Seu-Um, Aceh Besar, Indonesia. *C. gigantea* aqueous extract can be used as a bio-reductant for Ag^+^→Ag^0^ conversion, obtained by 48h incubation of Ag^+^, and the extract mixture in a dark condition. UV–Vis characterization showed that the surface plasmon resonance (SPR) peaks of AgNPs-leaf *C. gigantea* (AgNPs-LCg) and AgNPs-flower *C. gigantea* (AgNPs-FCg) appeared in the wavelength range of 410–460 nm. Scanning electron microscopy energy-dispersive X-ray spectrometry (SEM-EDS) revealed the agglomeration and spherical shapes of AgNPs-LCg and AgNPs-FCg with diameters ranging from 87.85 to 256.7 nm. Zeta potentials were observed in the range of −41.8 to −25.1 mV. The Kirby-Bauer disc diffusion assay revealed AgNPs-FCg as the most potent antimicrobial agent with inhibition zones of 12.05 ± 0.58, 11.29 ± 0.45, and 9.02 ± 0.10 mm for *Escherichia coli*, *Staphylococcus aureus*, and *Candida albicans*, respectively. In conclusion, aqueous extract from the leaves or flowers of *Calotropis gigantea* may be used in the green synthesis of AgNPs with broad-spectrum antimicrobial activities.

## 1. Introduction

Researchers have long studied plant extracts for their broad spectrum of medicinal properties such as anti-inflammatory [1], antibacterial [2,3], antifertility [4], termiticide, and nematicide activities [5]. Studies on plant extracts have recently gained more interest during the COVID-19 pandemic in the effort to find efficacious antiviral agents [6,7,8,9]. Notably, the bioactive compounds contained in plant extracts are strongly influenced by environmental factors [10]. For example, more diverse metabolites and higher-yield extracts were produced by plants that grow in coastlines and geothermal areas compared with those in other areas [11].

Several areas in Aceh Province have geothermal potential stemming from volcanic activities, including Mount Seulawah Agam, located in the Aceh Besar District [12,13]. Mount Seulawah Agam is a potential site for the construction of a geothermal power plant with an estimated energy capacity of 230 MW [14]. This mountain has several manifestations, namely Ie Brouk [15], Ie Seu-Um [16], and Ie Jue [17]. Bursts of mineral-rich water in each of these manifestations have spread to the surrounding environment, contributing to the mineral disposition in the soil, which subsequently affects the biosynthesis of plants therein. One of the plants thriving in the geothermal environment is *Calotropis gigantea,* which intensely grows in the area.

In a previous investigation, the ethanolic extract from the leaves of *C. gigantea* (collected from the manifestation of Ie Jue) was observed to have effective antibacterial activities against Gram-negative (*Porphyromonas gingivalis*) and Gram-positive bacteria (*Solobacterium moorei*) [18]. Another study witnessed that the lignan glycoside compounds contained in the latex of *C. gigantea* from the manifestation of Ie Brouk contained potential antiviral properties against influenza virus and anti-SARS-CoV-2 in silico [19]. Of the three aforementioned geothermal manifestations in Mount Seulawah Agam, *C. gigantea* is abundant in the Ie Seu-Um manifestation [20]. 

In a recent review, silver nanoparticles (AgNPs) were highlighted to possess strong antibacterial properties against Gram-negative and -positive bacteria, including the strains that have developed multidrug-resistant mechanisms [21]. Other than the common mechanism of the disintegrating bacterial cell wall membrane, AgNPs are uniquely capable of causing bacterial DNA and protein dysfunction by interacting with phosphorous or sulfur groups [22,23,24]. AgNPs may also induce apoptosis against bacterial cells by producing reactive oxygen species and free radicals, concomitant with the release of Ag^+^ from AgNPs and followed by a reaction with thiol groups [22,23,24]. Therefore, it is of importance to develop AgNPs as an efficacious antibacterial agent to curb the global health burden caused by bacterial infection, especially with the increasing trend of multidrug-resistant bacteria [25]. To achieve that, green synthesis using bioactive phytocompounds may enhance the bioactivity of AgNPs, as they possess the ability to overcome bacterial-drug-resistant mechanisms [21,26]. Green nanoparticle synthesis is defined as a means of obtaining metal nanoparticles with the help of bioreductor, plant, fungal, or bacterial extracts (produced using a green solvent, such as water). Due to the exclusion of toxic and ecotoxic reagents from the procedure, green synthesis is eco-friendly by nature and safe. 

Herein, we used flower and leaf extracts from *C. gigantea* collected from the Ie Seu-um geothermal manifestation to green synthesize AgNPs. Antimicrobial activities in *S. aureus, E. coli,* and *C. albicans* were tested to evaluate the bioactivity of the synthesized AgNPs. Multiple reports have been published regarding the green synthesis of silver nanoparticles [27,28]. However, research highlighting geothermal plants is currently underreported, hence the novelty of our research. Moreover, we conducted a longitudinal comparison between the extracts yielded from the flower and those from the leaf of *C. gigantea*; the results provide significant implications for the development of green nanoparticle synthesis research. 

## 2. Results and Discussion

### 2.1. Green Synthesis AgNPs-LCg and AgNPs-FCg

**Figure 1:**
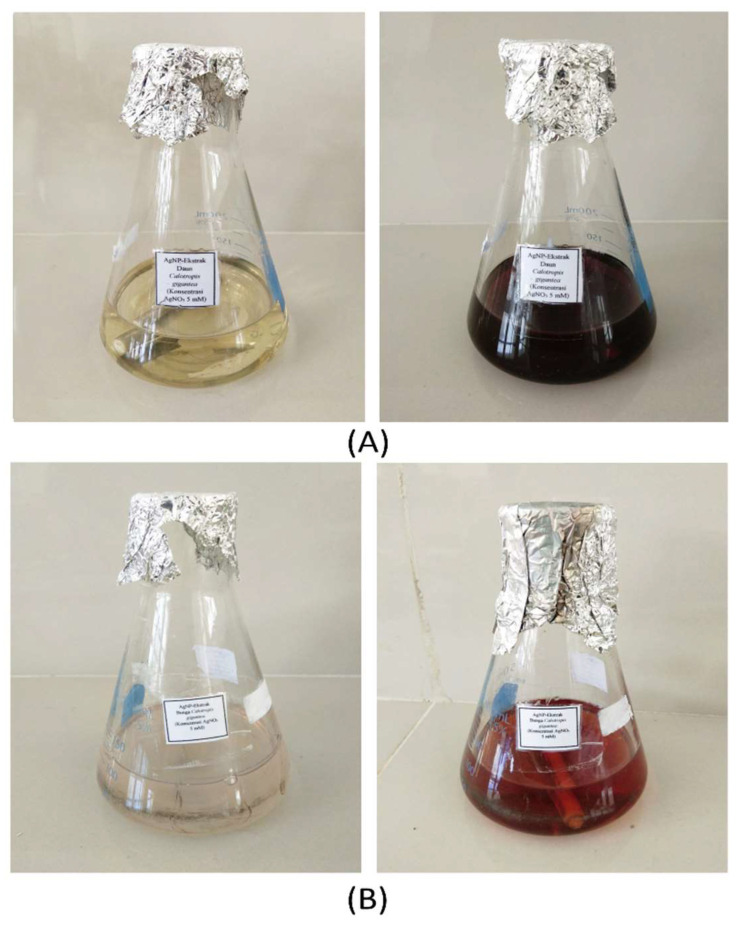
Visual appearance of AgNO_3_ solution mixed with leaves (**A**) and flower extract (**B**) from geothermal *C. gigantea* before (left) and after (right) the dark incubation.

The AgNPs produced from the leaves and flowers of *C. gigantea* were labeled as AgNPs-LCg and AgNPs-FCg, respectively. Color changing occurred and acted as an indicator of the Ag^+^→Ag^0^ reaction (Figure 1). In a mixture producing AgNPs-LCg, the transparent and yellowish solution turned into a blackish-brown solution following the incubation process. In the case of AgNPs-FCg, the colorless solution transformed into a yellow-brown solution (Figure 1). Similarly, previous reports have shown the color changing into yellow-brown [29,30,31,32] or dark brown [33,34,35,36,37,38] following the green synthesis of AgNPs.

The difference in color transformation between AgNPs-LCg and AgNPs-FCg could be attributed to the phytometabolite components contained in the leaves or flowers of the geothermal *C. gigantea*. Thus, we proceeded with our investigation on qualitative phytochemical screening, for which the data are presented in Table 1. Both extracts were positive for containing alkaloids, saponins, phenolics, and tannins. Flavonoids and terpenoids were detected in the flower extract but not in the leaf extract. Meanwhile, steroids were only observable in the leaf extract. 

### 2.2. Characterization of Silver Nanoparticles (AgNPs-LCg and AgNPs-FCg) 

#### 2.2.1. UV–Vis Spectrophotometry Analysis

UV–Vis spectrophotometers are rapid, easy-to-use, and sensitive tools frequently used for initial characterization of metal nanoparticles synthesized using biological methods [39]. The chromophore of secondary metabolites present in metal-reducing organic compounds could perform light absorption in UV and UV–Vis wavelength ranges [39]. Phytometabolites may act as electron donors and mediate the reduction of silver ions [33], thereby initiating surface plasmon resonance (SPR). This phenomenon is a complicated process defined as the excitation and coherent oscillation of electrons in an incoming electromagnetic field [39]. The absorption peak profile is dependent on the functional group and chromoionophore compositions of the plant extract and the extract concentration used in the AgNP synthesis [33].

**Figure 2:**
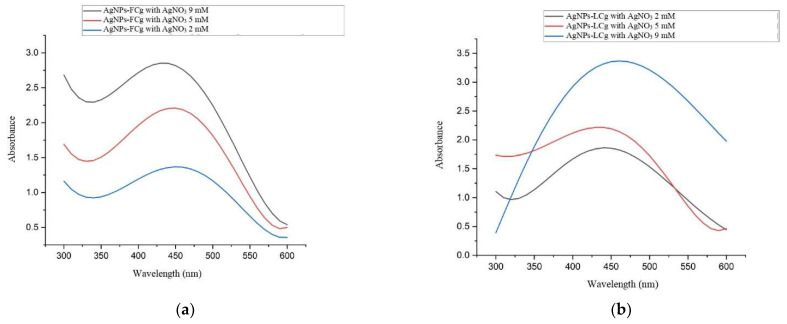
UV–Vis spectrophotometry characterization of (**a**) AgNPs-FCg and (**b**) AgNPs-LCg.

The SPR peaks of the AgNPs-FCg samples prepared using AgNO_3_ with concentrations of 2, 5, and 9 mM were found at 440, 450, and 460 nm, respectively (Figure 2). Meanwhile, the SPR of the AgNPs-LCg samples synthesized using AgNO_3_ with 2, 5, and 9 mM were found at 440, 430, and 460 nm, respectively (Figure 2). Our results do not deviate much in comparison with those previously reported [34,40]. As a comparison, a study preparing AgNPs using Indian *C. gigantea* flower reported an SPR peak at 422 nm [34]. In another study, UV–Vis characterization on AgNPs produced from *C. gigantea* leaf extract collected in the Shudqum region, Saudi Arabia, revealed an SPR peak at 450 nm [40].

#### 2.2.2. Fourier Transform Infrared (FTIR) Analysis

**Figure 3:**
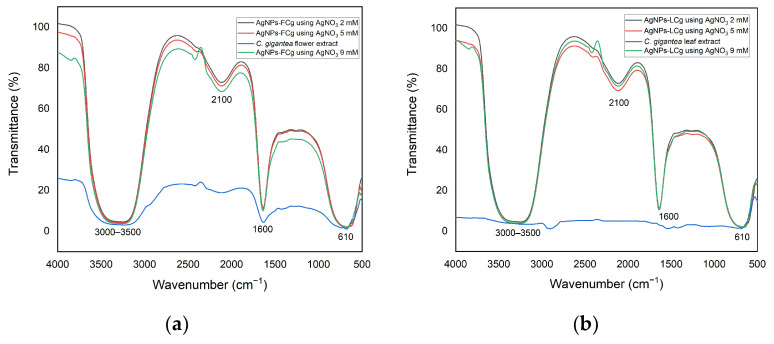
FTIR spectra of (**a**) AgNPs-FCg and (**b**) AgNPs-LCg at several concentrations of AgNO_3_.

We used Fourier transform infrared (FTIR) characterization to identify functional groups suspected to be involved in the AgNPs synthesis, for which the data ae presented in Figure 3. The spectral profiles of both AgNPs-FCg and AgNPs-LCg suggest the presence of O—H stretching, C=C stretching, N—H bending, and C—H bending vibrations assigned for the absorbance peaks observed at 3250, 2100, 1600, and 610 cm^−1^, respectively. A previous report suggested the involvement of the N—H functional group in AgNPs synthesis [41]. The absorption of O—H showed that polar compounds (likely flavonoids or phenolics) from plants (Table 1) are involved in the green synthesis of AgNPs. It was also claimed that the O—H and amine groups are components that function as capping agents to stabilize silver nanoparticles [42]. Our findings agree with previously reported AgNPs-LCg results showing that the presence of groups at 3421 and 2923 cm^−1^ are assigned to the bending vibration of N—H, 2853 cm^−1^ is assigned to the stretching vibration of C—H in alkanes, 2361 cm^−1^ indicates a primary amine group, 1457 cm^−1^ is assigned to the stretching vibration of protein, and 1116 cm^−1^ is assigned to the stretching vibration of C—O [40].

#### 2.2.3. SEM-EDS Analysis

**Figure 4:**
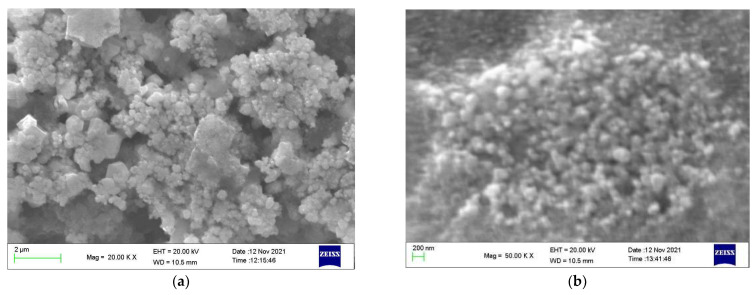
SEM image of AgNPs-LCg (**a**) and AgNPs-FCg (**b**) displayed at 20,000× and 50,000× magnification, respectively.

**Figure 5:**
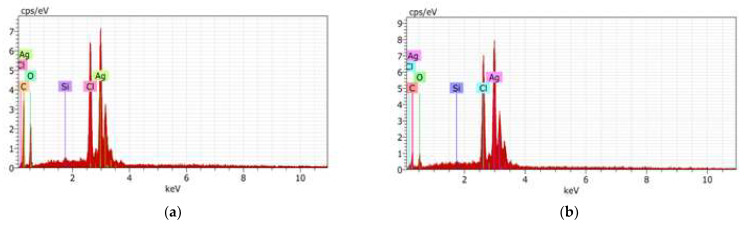
EDS spectra of (**a**) AgNPs-FCg and (**b**) AgNPs-LCg at several concentrations of AgNO_3_.

The scanning electron microscopy energy-dispersive X-ray spectroscopy (SEM-EDS) images of AgNPs-FCg and AgNPs-LCg surfaces are presented in Figure 4a,b, respectively. The formed AgNPs-FCg and AgNPs-LCg were observed to be agglomerated. In general, the morphology of formed AgNPs-FCg and AgNPs-LCg is spherical. The agglomeration of AgNPs synthesized from plants has been previously reported by those using the leaves of *C. gigantea* [40], *Andrographys paniculata, Phillanthus niruri, Tinuspora cordifolia* [29], *Nigella sativa* [43], *Brilliantaisia patula, Crossopteryx febrifuga*, *Senna siamea* [44], *Lampranthus coccineus, Malephora lutea* [35], *Platycodon grandiflorum* [30], *Memecylon umbellatum Burm F* [45], and *Malva parviflora* [33]. The EDS spectra of AgNPs-FCg and AgNPs-LCg, indicating the elements present in each sample, are presented in Figure 5. The list of elements of each sample obtained from the EDS analysis is provided in Figure 5. According to the results of the EDS analysis, metallic silver predominated among the elements observed in the sample. Therefore, this analysis confirmed that the produced particles were silver nanoparticles (AgNPs). Other elements are thought to be derived from secondary metabolites found in the *C. gigantea* plant that grows in mineral-rich geothermal areas.

#### 2.2.4. Zeta Potential Analysis

The results of the zeta potential analysis on AgNPs-LCg and AgNPs-FCg produced from the various concentrations of AgNO_3_ (2, 5, and 9 mM) are presented in Table 2. The synthesized AgNPs-LCg and AgNPs-FCg were relatively stable at −25.1 to −41.8 mV, respectively. Generally, the larger the negative zeta potential value, the more stable the AgNPs formed [30,46]. In this study, the concentration of AgNO_3_ as a metal precursor solution affected the stability of the produced AgNPs. In AgNPs-FCg, the stability increased as the concentration of AgNO_3_ increased. For AgNPs-LCg, the highest stability value was obtained when the AgNO_3_ concentration was 5 mM. 

The sizes of AgNPs-LCg and AgNPs-FCg obtained widely ranged: 163.5 ± 1.06–256.7 ± 2.82 and 87.85 ± 0.91–227.65 ± 0.07, respectively. The smallest size of the AgNPs was the AgNPs-LCg obtained when the [AgNO_3_] was 5 mM. The AgNPs size correlated with the SPR peak measured by UV−Vis spectroscopy, where the lower the SPR wavelength, the smaller the particle size obtained [47]. Therefore, in this present study, the particle size obtained through zeta potential analysis is corroborated by the earlier finding from UV−Vis spectroscopy, where the AgNPs-LCg produced from AgNO_3_ 5 mM had the lowest SPR wavelength. 

### 2.3. Antimicrobial Activity of Silver Nanoparticles (AgNPs-LCg and AgNPs-FCg)

One of the properties of AgNP that has long been a hot topic is its antimicrobial activity [34,48]. AgNP has attracted the interest of researchers because it has antibacterial, antifungal, antiviral, antiangiogenic, and cytotoxic properties against cancer cells that have not been replaced by the properties of other metals [39]. The exact mechanism by which AgNPs have toxic or antimicrobial activity remains a mystery and is a topic of debate [34]. Several studies have shown that the spherical shape, concentration, and type of silver nanoparticles may play a key role in the inhibition of bacteria, which are absorbed in bacterial cells to interact with bacterial DNA and proteins causing their damage, further resulting in more oxidative stress through the generation of reactive oxygen species (ROS). ROS accumulate into the mitochondrial membrane of bacteria, resulting in dysfunctional mitochondria, thereby inhibiting bacterial growth [49]. 

**Figure 6:**
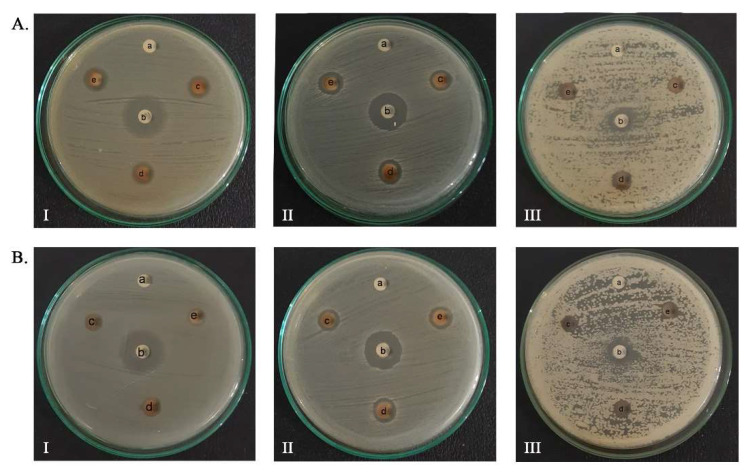
Antimicrobial activity analysis: (**A**) AgNPs-FCg; (**B**) AgNPs-LCg (I: *E. coli* bacteria, II: *S. aureus* bacteria, III: *C. albicans* fungus where a: negative control, b: positive control, c: sample with AgNO_3_ concentration of 2 mM, d: sample with AgNO_3_ concentration 5 mM, e: sample with 9 mM AgNO_3_ concentration).

In this study, antibacterial and antifungal evaluations of AgNPs-LCg and AgNPs-FCg were carried out using two bacterial pathogens (*E. coli* and *S. aureus*, which represented Gram-negative and -positive bacteria, respectively) and one fungal pathogen (*C. albicans)*. Images of the inhibition of the foregoing pathogens performed by AgNPs-LCg and AgNPs-FCg on Kirby-Bauer disc diffusion assays are presented in Figure 6. Based on the results obtained, the samples of AgNPs-LCg and AgNPs-FCg were found to inhibit the growth of bacteria and fungi as the inhibitory properties of bacterial and fungal pathogens increased in proportion to the increase in AgNO_3_ concentration in the synthesis process. The quantitative data of the inhibition zones are presented in more detail in Table 3. AgNPs-FCg had the highest antimicrobial activity in *S. aureus*, which was 12.05 ± 0.58 mm, followed by *E. coli* at 11.29 ± 0.45 mm and *C. albicans* at 9.02 ± 0.10 mm. Meanwhile, AgNPs-LCg had the highest antimicrobial activity in *S. aureus*, which was 10.60 ± 0.22 mm, followed by *C. albicans* at 8.90 ± 0.25 mm and *E. coli* at 8.40 ± 0.33 mm. 

The antibacterial activity of *S. aureus* was significantly different between the AgNPs-LCg and AgNPs-FCg inhibition zones. The AgNPs-LCg inhibition zone with 9 mM AgNO_3_ (which was the highest concentration in this study) was almost equivalent to the AgNPs-FCg inhibition zone with 2 mM AgNO_3_ (the lowest concentration in this study). In the *E. coli* antibacterial activity test, the AgNPs-LCg inhibition zone with AgNO_3_ 9 mM was lower than the AgNPs-FCg inhibition zone with AgNO_3_ 2 mM. This indicates that AgNPs-FCg is more potent in inhibiting the growth of Gram-positive and -negative bacteria than AgNPs-LCg isolated from the Ie Seu-um geothermal area. 

The difference in activity between AgNPs-LCg and AgNPs-FCg is thought to be due to differences in secondary metabolite components between the leaves and flowers (please revisit Table 1). The flower extract contained flavonoids, whereas the leaf extract did not. There are at least two possibilities for explaining this finding. Firstly, flavonoids in the flower extract facilitate the higher conversion of Ag^+^ into Ag^0^, attributed to their low redox potentials ranging from 0.23 to 0.75 V (while that of Ag is 0.80 V) [50]. Secondly, flavonoids are likely to bind with the Ag^0^ as a capping agent via a complex formation, as the compounds possess chelating moiety catechol [51]. Members of flavonoids were proven to inhibit bacterial growth by disrupting the cell wall membrane [52]. Therefore, as flavonoids bind to the AgNPs, synergistic inhibitory activities are expected.

It is also noteworthy that in our previous study, the methanol extract of the leaves and stems of *C. gigantea* growing in the Ie Seu-um area was reported to have no inhibition zone on the fungus *C. albicans* [20], but in this study, the antifungal activities against *C. albicans* by AgNPs-LCg and AgNPs-FCg samples were relatively high. With the minimum concentration of AgNO_3_ used (2 mM), AgNPs-FCg and AgNPs-LCg were able to inhibit the growth of this fungus by 7.52 ± 0.11 and 7.37 ± 0.29 mm, respectively. AgNO_3_ was reported to have the minimum inhibition against the foregoing microbes [53]. Taken altogether, the AgNPs significantly contributed to the antimicrobial activities of our samples. However, as a limitation of this present study, we did not conduct a direct comparison between the prepared AgNPs samples with the extracts and the AgNO_3_. Moreover, we did not use commercial AgNPs as a comparison.

## 3. Materials and Methods

### 3.1. Materials and Bioindicators

**Figure 7:**
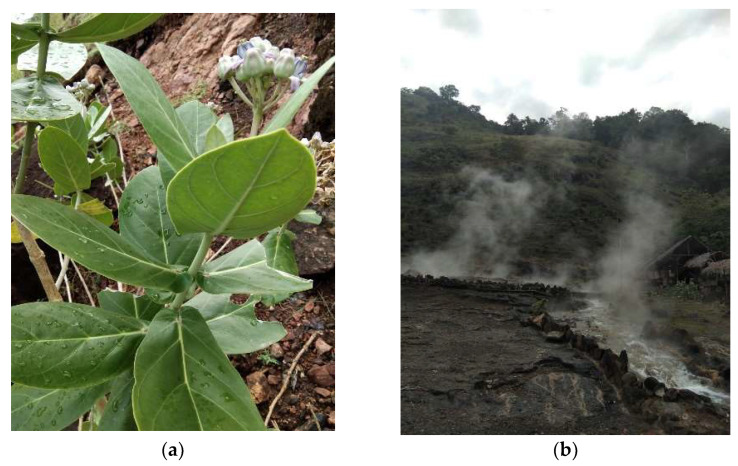
(**a**) *C. gigantea* plant; and (**b**) Ie Seu-Um geothermal manifestation area.

The samples used included the leaves and flowers of *C. gigantea* (Figure 7a) growing in the geothermal manifestation Ie Seu-Um, Aceh Besar, Aceh Province, Indonesia. The sampling locations was 5°32′50.97″ N, 95°32′55.10″ E at an altitude of 97 m above sea level (Figure 7b). 

The materials used in this study were AgNO_3_; Mayer’s, Dragendorff, Wagner, and Liberman-Burchard reagents; FeCl_3;_ Mg powder; gelatin; sulfuric acid; Mueller Hinton agar (MHA) media; and Sabarouds dextrose agar (SDA) purchased from Sigma Aldrich (St. Louis, MO, USA). Other materials used were distilled water, gentamicin, vancomycin, and ketoconazole. Meanwhile, the bacteria used as bioindicators in this study were *S. aureus* (ATCC 25923) and *E. coli* (ATCC 25922). The fungus used as a bioindicator was *C. albicans* (ATCC 10231). 

### 3.2. Plant Extraction

*C. gigantea* plants were taken and the flowers and leaves separated, then washed and cut into pieces before drying for two days. Sample extraction was carried out in different ways for both the flowers and leaves. The *C. gigantea* leaf extraction process was carried out by boiling 10 g of leaf sample in 100 mL of distilled water for 20 min and filtered using filter paper [40]. The *C. gigantea* flower was extracted by adding 200 mL of distilled water while stirring at room temperature for 15 min and filtering [34].

### 3.3. Phytochemical Test

Phytochemical analysis was carried out to examine the contents of alkaloids, saponins, phenolics, tannins, flavonoids, steroids, and terpenoids in the flower and leaf extracts of *C. gigantea*. Phytochemical screening followed the standard method [54]. The alkaloid test used Mayer, Dragendorff, and Wagner reagents. The saponin test was carried out by examining the stable foam after being shaken using distilled water. The steroid test and terpenoid test used Liberman-Burchard reagent. The phenolic test used FeCl_3_ and flavonoid test Mg powder. As for the tannin test, gelatin and sulfuric acid were used. 

### 3.4. Green Synthesis of AgNPs

**Figure 8:**
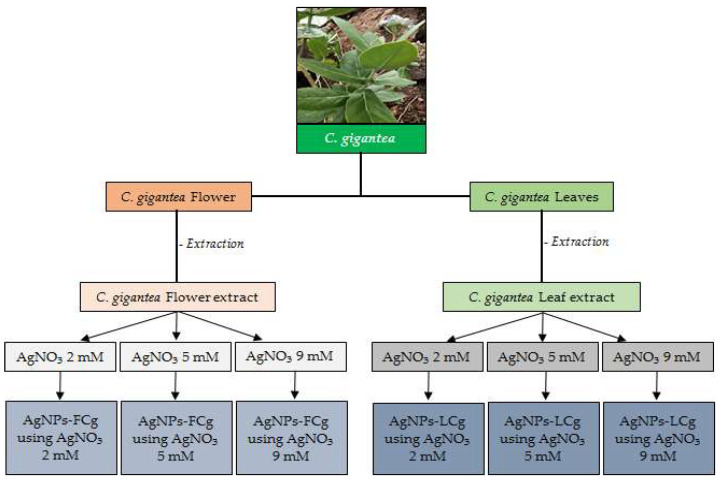
Schematic diagram of the green synthesis of AgNPs-LCg and AgNPs-FCg.

The synthesis of silver nanoparticles using *C. gigantea* leaf extract (AgNPs-LCg) was carried out by mixing 90 mL AgNO_3_ (2, 5, and 9 mM, separately) with 10 mL of *C. gigantea* leaf extract (Figure 8). The reaction was incubated at room temperature (25 ± 1 °C) under constant stirring using a magnetic stirrer at 60 rpm for 48 h in the dark condition [55]. The synthesis of silver nanoparticles using *C. gigantea* flower extract (AgNPs-FCg) was carried out with the same procedure. We then observed the color formed from the reaction. The results of the AgNPs-LCg and AgNPs-FCg reactions were measured for absorbance using UV–Vis spectrophotometry (Shimadzu, UV 2500, Shimadzu Suzhou Instruments Mfg. Co., Ltd., Jiangsu, China) at a wavelength of 300–600 nm and centrifuged (Nuve, NF 800R model, Ankara, Turkey) at 13,000× *g* for 10 min.

### 3.5. Characterization of Silver Nanoparticles (AgNPs-LCg and AgNPs-FCg)

AgNPs-LCg and AgNPs-FCg were characterized using Fourier transform infrared (FTIR) spectroscopy (Search 630 FTIR Spectrometer, Agilent Technologies, Santa Clara, CA, USA), and scanning electron microscopy energy-dispersive x-ray spectroscopy (SEM-EDX, Carl Zeiss-Bruker EVO MA 10, Carl Zeiss Microscopy, White Plains, NY, USA). The characterization of the zeta potential value (mV) and polydispersity index (PI) of samples were analyzed using a Zetasizer Nano (Horiba SZ-100, Horiba Mfg. Co., Ltd., Kyoto, Japan). 

### 3.6. Antimicrobial Activity Assay

Antibacterial and antifungal tests were performed using the Kirby-Bauer disc diffusion method. The AgNPs-LCg and AgNPs-FCg were tested for their inhibition zones on colonies of pathogenic bacteria *S. aureus* and *E. coli*, and the fungus *C. albicans*. Both the bacterial colonies were grown in an MHA medium for 24 h at 37 °C. Furthermore, the colonies from the liquid medium were spread on a Petri dish containing MHA agar using a spreader. Prepared sterile paper discs (6 mm in size) were placed on the Petri dishes used for inoculation. AgNPs-LCg and AgNPs-FCg samples with various concentrations (2, 5, or 9 mM) were then loaded onto each paper disc. The positive control used for the Gram-positive antibacterial assay was vancomycin, while the positive control for the Gram-negative antibacterial assay was gentamicin. The inhibitory diameter was measured after incubation at 24 h (37 °C). The antifungal activity of *C. albicans* was also carried out with the same method but the medium used was SDA media, and the positive control used for the antifungal assay was ketoconazole. The assays were prepared in triplicate. 

## 4. Conclusions

We successfully carried out green synthesis of AgNPs using leaf and flower extracts of *Calotropis gigantea*, which grows in the geothermal manifestation Ie Seu-Um, Aceh Besar, Indonesia. Using AgNO_3_ as a metal precursor with concentrations of 2, 5, and 9 mM, it is known to form SPR peaks in the 410–460 nm range. The FTIR results also showed the functional groups detected in AgNPs-FCg and AgNPs-LCg. In addition, AgNPs-FCg and AgNPs-LCg have a sphere-shaped morphology, good stability, and antimicrobial activity against Gram-positive bacteria, Gram-negative bacteria, and fungi. AgNPs-FCg is known to have a larger inhibition zone than AgNPs-LCg. As a result of the findings regarding nanoparticle size, we recommend additional research into reaction process optimization to reduce the generally accepted nanoparticle size to values lower than those found in this study.

## Figures and Tables

**Table 1:** Qualitative phytochemical analysis of AgNPs-FCg and AgNPs-LCg.

Secondary Metabolites	AgNPs-FCg	AgNPs-LCg
Saponins	+(ve)	+(ve)
Phenolic	+(ve)	+(ve)
Tannins	+(ve)	+(ve)
Flavonoids	+(ve)	−(ve)
Terpenoids	+(ve)	−(ve)
Steroids	−(ve)	+(ve)
Alkaloids	+(ve)	+(ve)

Note: +(ve): positive; −(ve): negative.

**Table 2:** Zeta potential analysis parameters of AgNPs-LCg and AgNPs-FCg.

[AgNO_3_]	AgNPs-FCg (Mean ± SD)		AgNPs-LCg (Mean ± SD)	
	Stability (mV)	Size (nm)	Stability (mV)	Size (nm)
2 mM	−33.05 ± 0.00	256.7 ± 2.82	−40.5 ± 0.56	227.65 ± 0.07
5 mM	−30.3 ± 0.00	200.8 ± 0.14	−41.8 ± 0.14	87.85 ± 0.91
9 mM	−25.1 ± 0.00	163.5 ± 1.06	−31.35 ± 0.7	188.35 ± 3.32

**Table 3:** Antimicrobial activities of AgNPs-FCg and AgNPs-LCg.

Sample	Concentration of [AgNO_3_] (mM)	Inhibition Zone, Mean ± SD (mm)		
		*Staphylococcus* *aureus*	*Escherichia* *coli*	*Candida* *albicans*
AgNPs-FCg	2	10.53 ± 0.57	9.89 ± 0.56	7.52 ± 0.11
	5	11.24 ± 0.83	10.54 ± 0.59	8.12 ± 0.16
	9	12.05 ± 0.58	11.29 ± 0.45	9.02 ± 0.10
AgNPs-LCg	2	10.10 ± 0.08	7.85 ± 0.18	7.37 ± 0.29
	5	10.48 ± 0.23	8.18 ± 0.13	7.95 ± 0.26
	9	10.60 ± 0.22	8.40 ± 0.33	8.90 ± 0.25
Control		17.74 ± 0.28 ^a^	19.45 ± 0.69 ^b^	10.20 ± 0.12 ^c^

Control: ^a^ vancomycin; ^b^ gentamicin; ^c^ ketoconazole.

## Data Availability

Data that support the findings of this study are available from the corresponding author upon reasonable request.

## Sample Availability

Samples of the compounds are available from the authors.
